# Learning curve of trans‐areola single‐site endoscopic thyroidectomy in a high‐volume center: A CUSUM‐based assessment

**DOI:** 10.1002/cam4.6307

**Published:** 2023-07-03

**Authors:** Ling Zhan, Ming Xuan, Hao Ding, Juyong Liang, Qiwu Zhao, Lingxie Chen, Zheyu Yang, Xi Cheng, Jie Kuang, Jiqi Yan, Wei Cai, Weihua Qiu

**Affiliations:** ^1^ Department of General Surgery, Ruijin Hospital Shanghai Jiao Tong University School of Medicine Shanghai China; ^2^ Department of General Surgery, Ruijin Hospital Gubei Campus Shanghai Jiao Tong University School of Medicine Shanghai China

**Keywords:** CUSUM analysis, learning curve, single‐site endoscopic thyroidectomy, trans‐areola approach

## Abstract

**Background:**

Limited attempts have been made in trans‐areola single‐site endoscopic thyroidectomy (TASSET) due to technical challenges and the lengthy time for proficiency. This study aimed to define the learning curve of TASSET and to describe improvements in operative performance over time.

**Methods:**

Based on 222 consecutive TASSET procedures, the learning curve was established according to the operation time by using cumulative sum analysis (CUSUM). The end‐point of learning curve was defined as the number of cases necessitated to reach the initial surgical proficiency stage. The demographic information, surgical and oncological outcomes, surgical stress, and postoperative complications were also analyzed.

**Results:**

There were 70 cases of simple lobectomy for benign nodules and 152 cases of lobectomy with central neck dissection (CND) for malignancy. The mean operative time was 106.54 ± 38.07 min (range: 46–274 min). The learning curve identified two phases: the skill acquisition phase (Case 1–Case 41) and the proficiency phase (Case 42–Case 222). There were no significant differences in demographic information, drainage amount and duration, oncological outcomes, and postoperative complications between the two phases (*p* > 0.05). Both operation time and postoperative hospitalization decreased significantly in Phase 2 (154.63 ± 52.21 vs. 95.64 ± 22.96 min, *p* < 0.001; 4.12 ± 0.93 vs. 3.65 ± 0.63 days, *p* < 0.001). Additionally, the mean variations of surgical stress factors (C‐reactive protein and erythrocyte sedimentation rate) decreased significantly as the phase progress. The case number required for proficiency phase in benign and malignant tumor were 18 and 33, respectively, and lymph node resection posed a significant impact on the endpoint of the learning curve (*p* < 0.001). Meanwhile, the size of nodule showed no significant impact (*p* = 0.622). For right‐handed surgeons, 16 cases and 25 cases were required for technical competence in left‐sided and right‐sided lesions, respectively, and no significant difference reached (*p* = 0.266).

**Conclusions:**

TASSET has demonstrated safe and technically feasible with comparable oncological outcomes. Experience of 41 cases was required for surgical competence and proficiency. The initial learning stage could be more quickly adopted by high‐volume thyroid surgeons with standardized procedures.

## Graphical abstract

The present study is the first one to evaluate the learning curve of trans‐areola single‐site endoscopic thyroidectomy (TASSET) in terms of operation time and surgical stress. Experience of 41 cases was required for surgical competence and proficiency in TASSET and benign nodule cases were recommended for the learning stage.

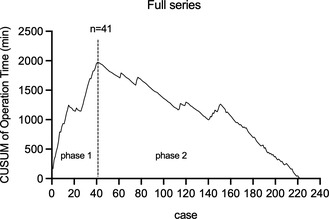

## 1 INTRODUCTION

Given the increasing number of thyroidectomies in past decade, a growing cosmetic desire has been witnessed from both patients and surgeons to avoid scar in anterior cervical neck area.^1^, ^2^, ^3^, ^4^ Conventional open thyroidectomy is currently the standardized approach for thyroid diseases with reliable radicality and safety, and this procedure results in a conspicuous scar on the neck inevitably.^5^ Since the first report of endoscopic parathyroidectomy,^6^ remote‐accessed endoscopic approach has been progressively attempted in neck surgery.^7^, ^8^, ^9^, ^10^ However, there is still doubt that multiple‐site endoscopic thyroidectomy could incur potentially higher surgical burden and postoperative discomfort with limited cosmetic effects.^11^, ^12^, ^13^

On the basis of oncological radicality and safety, as proposed by the evidence‐based practices of enhanced recovery after surgery,^14^ real minimal invasion also plays a critical part in enhanced recovery.^15^ Trans‐areolar single‐site endoscopic thyroidectomy (TASSET) only leaves a tiny unilateral incision and minimizes the subcutaneous dissection area.^11^, ^13^ Nevertheless, due to technical challenges, this practice is rarely attempted, and only a limited number of cases have been reported.^16^ Evidenced by a case‐match study with large‐scale population, the oncological radicality and safety of thyroidectomy for thyroid cancer via TASSET were validated in our previous study with pronounced cosmetic advantages.^13^ To overcome the technical obstacles in TASSET, it is important to have a basic comprehension of standard surgical procedure and prevent avoidable complications. In this study, to assess the learning curve for TASSET accurately and comprehensively, the cumulative sum (CUSUM) analysis of operation time was performed in chronological order. In addition to analyze the basic concerns about the lengthy time to proficiency, defining the learning curve can not only establish teaching curriculums but also describe evolution of TASSET technique. More importantly, it can enable to visualize the modifications of standard procedures in TASSET for benign and malignant cases with different nodule size.

## 2 METHODS

### 2.1 Patient inclusion and exclusion

The inclusion criteria were (1) patients younger than 70 years old; (2) according to thyroid imaging reporting and data system (TI‐RADS) grading, thyroid nodule(s) classified as level III in ultrasonography, but no more than 5 cm; (3) thyroid nodule(s) identified as differentiated thyroid carcinoma (DTC) by fine needle aspiration biopsy (FNAB); (4) according to the 8th American Joint Committee on Cancer (AJCC) staging system,^17^ the primary T stage in DTC patients was not higher than T2, the lymph node (LN) stage was cN0 or cN1a and there was no distant metastasis (M0); (5) patients exhibited high cosmetic expectations; (6) patients tended to have scar diathesis. The exclusion criteria were: (1) history of neck surgery or radiation; (2) LNs were fused with each other or fixed in the neck; (3) fixed vocal cord was confirmed with laryngoscopy before surgery; (4) extrathyroidal invasion or involvement, cN1b lymph metastases or distant metastases preoperatively.

### 2.2 Patient recruitment

From October 2013 to June 2018, 222 eligible consecutive patients were recruited for the current retrospective study at our center. All TASSETs were performed by the same surgeon. This study was approved by the Ethics Committee and the Institutional Review Board of Shanghai Ruijin Hospital (Ruijin LL‐14‐2006). All patients were notified of the TASSET procedures and were conscious of the potential benefits and risks. All patients signed the informed consent form as the agreement for undergoing the operation and the use of the data we collected perioperatively. This work has been reported in line with the STROCSS criteria^18^ and has been registered at http://www.researchregistry.com (UIN: researchregistry5132).

### 2.3 Surgical procedure

**FIGURE 1:**
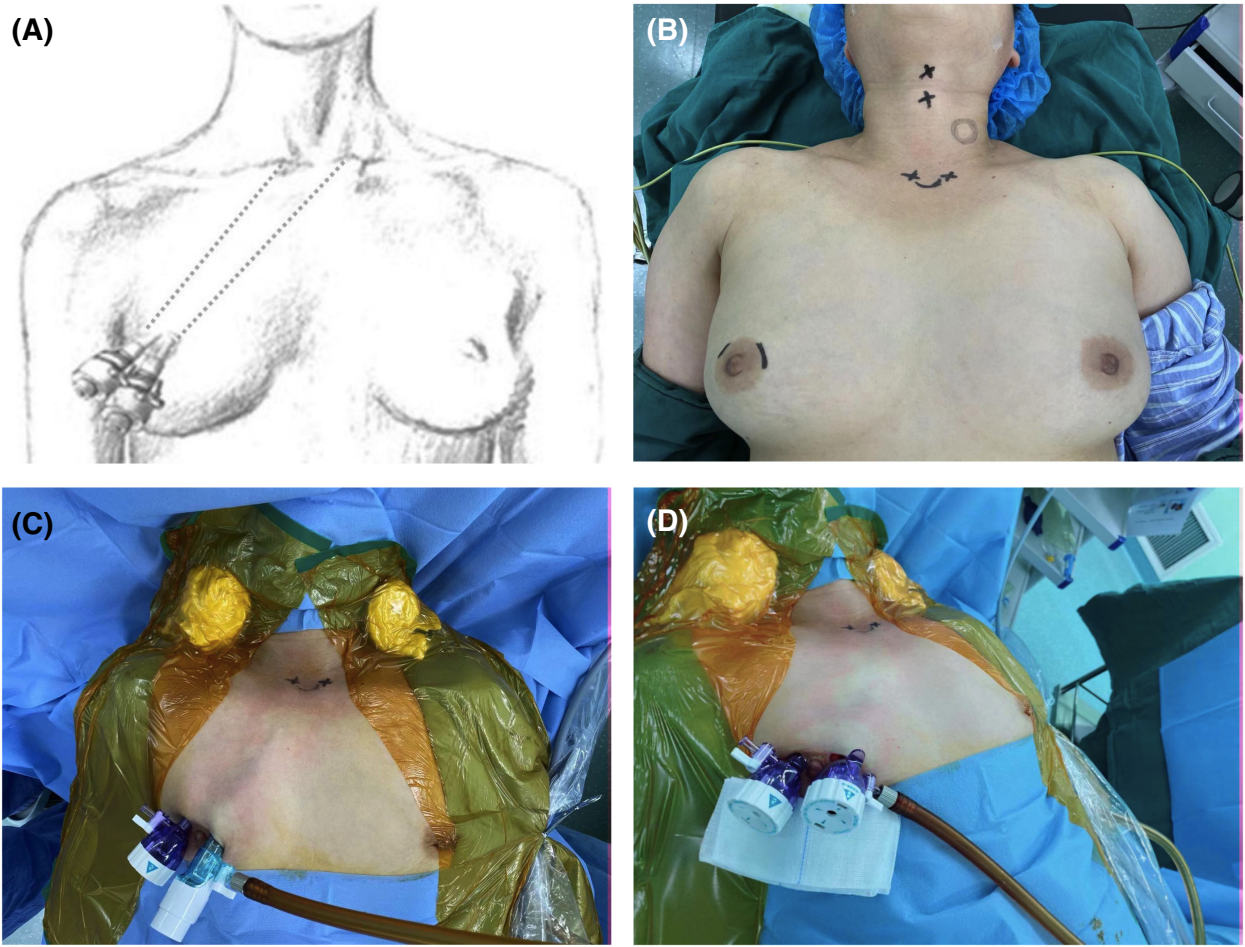
Surgical approach via trans‐areola single‐site endoscopic thyroidectomy (TASSET). (A) The schematic diagram of TASSET approach. (B) One 12‐mm and one 5‐mm incisions were made at 11 o'clock and 2 o'clock of the mammary areola, respectively. (C) and (D) The placement of trocars during surgery.

**FIGURE 2:**
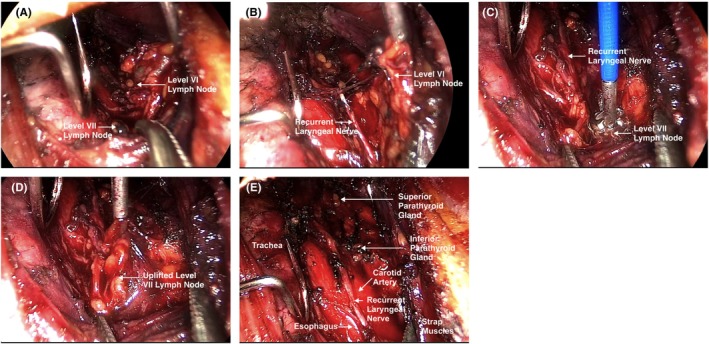
Clearance of central nodal tissues. (A) The exposure of central lymph node. (B–D) Upon the identification and protection of recurrent laryngeal nerve, Level VI and Level VII dissection were accomplished. (E) The scheme of surgical field after central neck dissection.

In our previous study, we have explored the standard surgical procedure. Briefly, all TASSETs were performed using the endoscopic technique through trans‐areola incisions. One 12‐mm incision and one 5‐mm incision were made at 11 o'clock and 2 o'clock of the contralateral side mammary areola of the tumor, respectively (Figure 1). Approximately thirty milliliters of tumescence were administered between the superficial and deep layers of the superficial fascia in front of the manubrium. The 12‐mm trocar was inserted with a facedown 30° endoscope, and the 5‐mm trocar was fitted with one operating apparatus equipped with Ultracision harmonic scalpel (Harmonic ACE, HAR23; Ethicon Endo‐Surgery, LLC) (Figure 1). With the aid of needle retractors and mini‐clamps, thyroid resection was implemented upon identification and careful protection of the recurrent laryngeal nerve (RLN) and parathyroid gland. Central neck dissection (CND) was performed in cases of malignant tumors detected by FNAB preoperation or rapid frozen section examination intraoperation (Figure 2). One 3‐mm drainage tube was placed in situ for indicated patients after careful hemostasis, followed by the closure of the linea alba cervicalis and the trans‐areola incisions.^13^

### 2.4 Perioperative parameters

Blood tests were measured routinely, including the serum concentrations of thyroid functional parameters and calcium level. In addition, inflammatory factors, including erythrocyte sedimentation rate (ESR) and C‐reactive protein (CRP), immunologic parameters (IgA, IgG, IgM, and IgE), and complements (C3, C4, and CH50) were examined 2 days before and 1 day after surgery. All patients went through high‐resolution staging ultrasonography and were classified according to the TI‐RADS system. The cervical LNs were regionally detected as well. The malignancy of lesions was determined preoperatively on the basis of FNAB cytologic examinations. Contrasted CT was performed on patients with possible tumor infiltration to better identify tumor invasion, bulk and indiscernible LNs. For each patient, the mobility of the vocal cord was assessed by indirect laryngoscopy preoperatively.^13^, ^19^

### 2.5 Surgical and oncologic outcomes

The main surgical outcomes involved operation time, blood loss, drainage amount and duration, postoperative hospitalization, and intraoperative and postoperative complications. Operation time was defined as the moment between the incision and closure of the skin. Blood loss was estimated accurately by dry medical gauze strips (0.5 × 5 cm). The occurrences of complications were also documented. The intraoperative operative morbidities principally involved vascular and RLN injury. The postoperative complications consisted of RLN injury, seroma, hematoma, hypocalcemia, and flap burning. Fiberoptic laryngoscopy was employed to assess vocal cord mobility in patients complaining of dyspnea or hoarseness, and cord paralysis was defined as a RLN injury.^13^, ^20^, ^21^ Permanent RLN paralysis was considered when there was no evidence of recovery within 12 months. Flap seroma or hematoma was documented in terms of neck swelling and tenderness.^13^ Hypocalcemia was defined when the ionized plasmatic calcium was lower than normal range and low parathyroid hormone.

The oncologic measurements included pathological results, retrieval of LNs, and cancer recurrence. The numbers of total LNs and metastatic LNs were counted based on pathological examination. The TNM stage was verified in accordance with the 8th AJCC system.^17^ A repeat ultrasonography for recurrence estimation was performed every 6 months after surgery. Recurrence was suspected with the presence of de novo nodules greater than 3 mm in the former thyroid bed and LNs in the cervical region and was further proved by FNA.^13^

### 2.6 Statistical analysis

CUSUM method was performed to quantitatively analyze the learning curve.^22^, ^23^, ^24^ All cases were evaluated in terms of operation time in chronological order to calculate the CUSUM.^25^ As for the first case, the CUSUM value represented the difference between the operation time of the first case and the overall average operation time. For the second case, the CUSUM value was the sum of the previous case's CUSUM and the difference between the second case operation time and the overall average operation time. This process continued to the last case.^26^, ^27^, ^28^, ^29^, ^30^

For quantitative variables, the results are presented as the mean ± SD. For the analysis of continuous and categorical data, student's *t*‐test and Chi‐squared analysis were applied, respectively. The statistical analyses were conducted using IBM SPSS Statistics software, version 23, and *p* < 0.05 was considered statistically significant.

## 3 RESULTS

### 3.1 Demographics information

During the period of study, 222 patients were enrolled. The mean age was 38.85 ± 10.71 years (range: 20–68 years) and 181 (81.5%) patients were female. 118 (53.2%) cases were right‐sided lesions. The number and size of the nodule were also documented. For those patients with multiple foci, the size was calculated as the summation of the lengths of all foci. The benign nodules had a mean size of 2.68 ± 0.56 cm (range: 2.0–4.0 cm), the malignant nodules had a mean size of 0.66 ± 0.50 cm (range: 0.1–3.2 cm) and 11.7% (26 of 222) cases were multifocal (Table 1).

**TABLE 1:** Demographic characteristics.

Characteristics	Full series (*n* = 222)	Phase 1 (*n* = 41)	Phase 2 (*n* = 181)	*p*
Age (mean ± SD, years)	38.85 ± 10.71	39.20 ± 10.71	38.77 ± 10.74	0.821
Sex (*n*)				0.849
Male	41	8	33	
Female	181	33	148	
BMI (mean ± SD, kg/m^2^)	23.37 ± 3.16	23.21 ± 3.43	23.40 ± 3.11	0.733
Lesion size (mean ± SD, cm)				
Benign	2.68 ± 0.56	2.48 ± 0.34	2.70 ± 0.58	0.130
Malignant	0.66 ± 0.50	0.78 ± 0.51	0.62 ± 0.50	0.111
Lesion side (*n*)				0.266
Left	104	16	88	
Right	118	25	93	
Number of tumor foci (*n*)				0.085
Solitary	196	33	163	
Multifocal	26	8	18	

Abbreviation: BMI, body mass index.

### 3.2 Surgical profile

70 cases of simple lobectomy for benign nodules and 152 cases of lobectomy with CND for malignancy were performed via TASSET. The mean operative time was 106.54 ± 38.07 min. The estimated intraoperative blood loss was less than 10 mL by using dry medical gauze strips. Drainage placement was indicated for 38 patients, the drainage volume was 32.03 ± 15.23 mL and the total placement duration was 1.71 ± 0.57 days. And the length of postoperative hospitalization was 3.73 ± 0.72 days (Table 2).

**TABLE 2:** Surgical and oncological profiles.

Variables	Full series	Phase 1	Phase 2	*p*
	Case 1–222	Case 1–41	Case 42–222	
Conversion to open (*n*)	0	0	0	>0.999
Operation time (mean ± SD, min)	106.54 ± 38.07	154.63 ± 52.21	95.64 ± 22.96	<0.001
Drainage volume (mean ± SD, mL)	32.03 ± 15.23	29.09 ± 17.58	33.22 ± 14.35	0.456
Drainage duration (mean ± SD, days)	1.71 ± 0.57	1.45 ± 0.52	1.81 ± 0.56	0.074
Postoperative hospitalization (mean ± SD, days)	3.73 ± 0.72	4.12 ± 0.93	3.65 ± 0.63	<0.001
Pathology (*n*)				0.159
Benign	70	8	62	
PTC	151	33	118	
FTC	1	0	1	
Capsular invasion (*n*)	0	0	0	>0.999
Extrathyroidal extension (*n*)	0	0	0	>0.999
pT stage (*n*)				0.099
1a	128	24	104	
1b	20	8	12	
2	4	1	3	
pN stage (*n*)				0.215
0	79	14	65	
1a	73	19	54	
AJCC TNM stage (*n*)				0.297
I	147	31	116	
II	5	2	3	
III	0	0	0	
Total number of LNs retrieved (mean ± SD, *n*)	3.14 ± 1.75	3.15 ± 1.70	3.14 ± 1.78	0.980
Number of metastatic nodes (mean ± SD, *n*)	1.05 ± 1.37	1.00 ± 1.15	1.06 ± 1.43	0.806
Cancerous recurrence (*n*)	0	0	0	>0.999

Abbreviations: FTC, papillary thyroid carcinoma; LNs, lymph nodes; PTC, papillary thyroid carcinoma.

### 3.3 Oncologic outcomes

The pathological analysis indicated that 152 malignant cases was composed of 151 cases of papillary thyroid carcinoma (PTC) and one case of follicular thyroid carcinoma (FTC). In accordance with the 8th AJCC TNM stage system, 147 cases were stage I and five cases were stage II. In detail, 13 patients were over the age of 55 years: eight patients had a stage I case, and five patients had a stage II case. All the remaining 139 patients younger than 55 years old were diagnosed with stage I. No cases of capsular invasion or extrathyroidal extension were observed. The mean retrieved LN count was 3.14 ± 1.75 (range: 2–10) while the mean metastatic LN count was 1.05 ± 1.37 (range: 0–7). No cases of recurrent lesions or residual parenchyma in the thyroid bed were observed during the follow‐up period (Table 2).

### 3.4 Surgical stress

Inflammatory factors (CRP and ESR), immunologic parameters (IgA, IgG, IgM, and IgE), and complements (C3, C4, and CH50) were examined. The variations were calculated by preoperative and postoperative parameters. The mean variations of traumatic factors (CRP and ESR) were 2.77 ± 2.56 mg/L and 2.90 ± 2.44 mm/h. The mean variations of IgA, IgG, IgM, and IgE were 0.30 ± 0.42 g/L, 0.87 ± 0.69 g/L, 0.10 ± 0.10 g/L, and 2.08 ± 1.66 IU/mL, respectively. The mean variations of C3, C4, and CH50 were 0.10 ± 0.13 g/L, 0.03 ± 0.03 g/L and 2.33 ± 2.18 U/mL (Table 3).

**TABLE 3:** Evaluation of surgical stress.

Variables	Full series	Phase 1	Phase 2	*p*
	Case 1–222	Case 1–41	Case 42–222	
ΔCRP (mean ± SD, mg/L)	2.77 ± 2.56	4.29 ± 3.34	2.28 ± 2.08	0.022
ΔESR (mean ± SD, mm/h)	2.90 ± 2.44	4.31 ± 2.46	2.50 ± 2.31	0.017
ΔIgE (mean ± SD, IU/mL)	2.08 ± 1.66	2.15 ± 1.74	2.06 ± 1.66	0.870
ΔIgA (mean ± SD, g/L)	0.30 ± 0.42	0.41 ± 0.45	0.27 ± 0.41	0.301
ΔIgG (mean ± SD, g/L)	0.87 ± 0.69	1.16 ± 0.78	0.79 ± 0.66	0.106
ΔIgM (mean ± SD, g/L)	0.10 ± 0.10	0.09 ± 0.07	0.10 ± 0.11	0.748
ΔC3 (mean ± SD, g/L)	0.10 ± 0.13	0.09 ± 0.07	0.10 ± 0.15	0.917
ΔC4 (mean ± SD, g/L)	0.03 ± 0.03	0.03 ± 0.03	0.03 ± 0.03	0.756
ΔCH50 (mean ± SD, U/mL)	2.33 ± 2.18	2.54 ± 2.36	2.27 ± 2.15	0.700

### 3.5 Safety evaluation

During operation, no severe vascular morbidities were noted. Due to unidentifiable adhesion, partial RLN fibers were accidentally injured in the plane where the RLN entered the larynx in two cases. The nerve was successfully sutured in an end‐to‐end anastomosis using 6–0 Prolene sutures (ETHICON). These two patients were considered permanent cord palsy. Additionally, 11 patients developed transient voice hoarseness postoperatively. These cases showed improvement gradually and were asymptomatic after 3.18 ± 1.17 months. No patients experienced hypocalcemia. One case was complicated with flap burning in anterior neck area. Postoperative flap seroma or hematoma occurred in 12 patients. None required subsequent surgical intervention, and most of them were treated with observation and local compression (Table 4).

**TABLE 4:** Safety evaluation.

Variables	Full series	Phase 1	Phase 2	*p*
	Case 1–222	Case 1–41	Case 42–222	
Intraoperative morbidity (*n*)				
Vascular injury	0	0	0	>0.999
RLN injury	2	1	1	0.336
Postoperative cord palsy (*n*)				
Transient	11	2	9	>0.999
Permanent	2	1	1	0.336
Recovery duration of RLN (mean ± SD, months)	3.18 ± 1.17	3.00 ± 1.41	3.22 ± 1.20	0.822
Hypocalcemia (*n*)	0	0	0	>0.999
Postoperative flap seroma/hematoma (*n*)				
Observation	12	3	9	0.828
Bedside aspiration	0	0	0	>0.999
Flap burning (*n*)	1	1	0	0.185

### 3.6 Learning curve and surgical improvements

**FIGURE 3:**
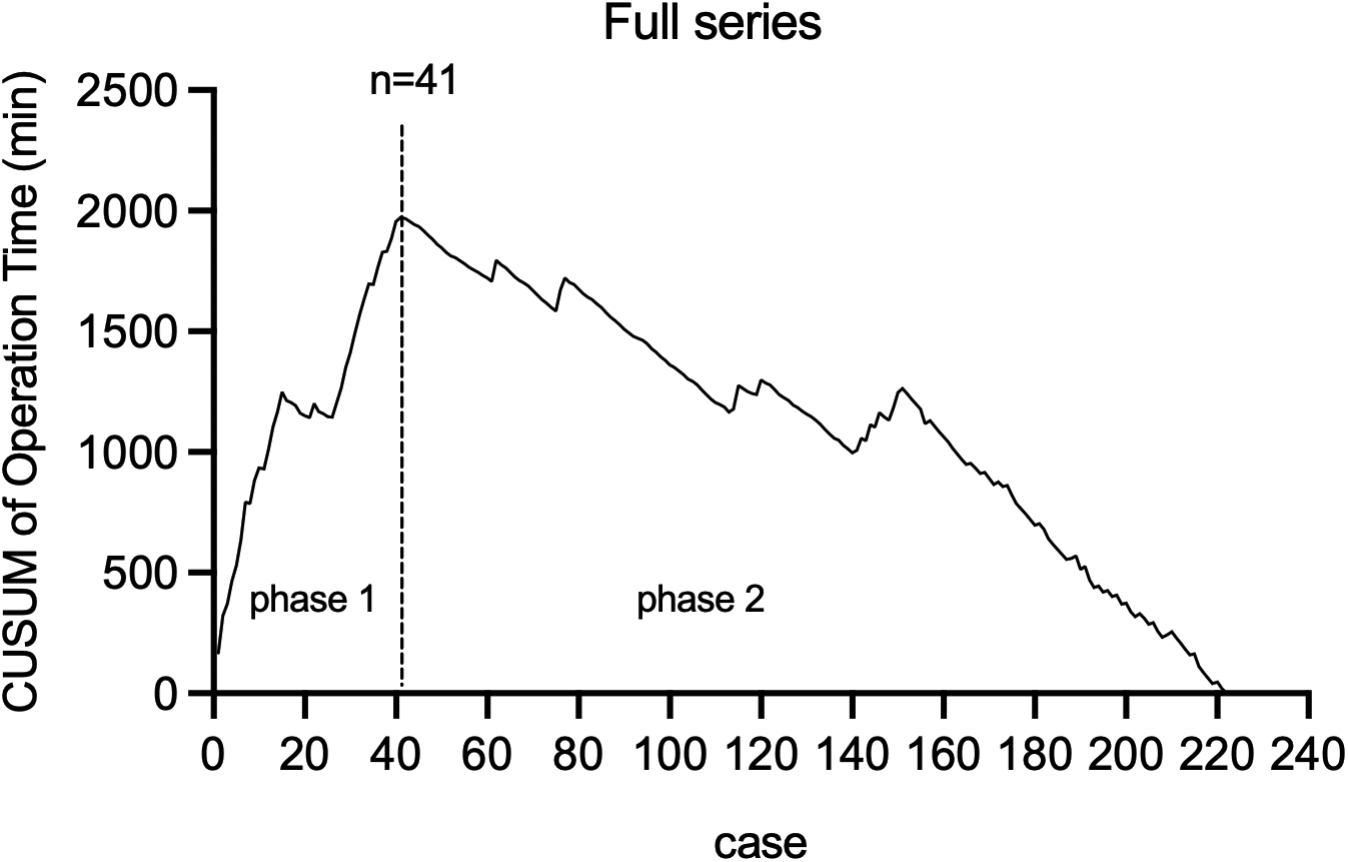
The cumulative sum (CUSUM) curve of operation time for the full series. The vertical line refers to the turning point at which the surgeon transitioned from the skill acquisition phase to the proficiency phase. The turning point is at Case 41. The operation time significantly improved from 154.63 ± 52.21 min (Phase 1) to 95.64 ± 22.96 min (Phase 2).

**FIGURE 4:**
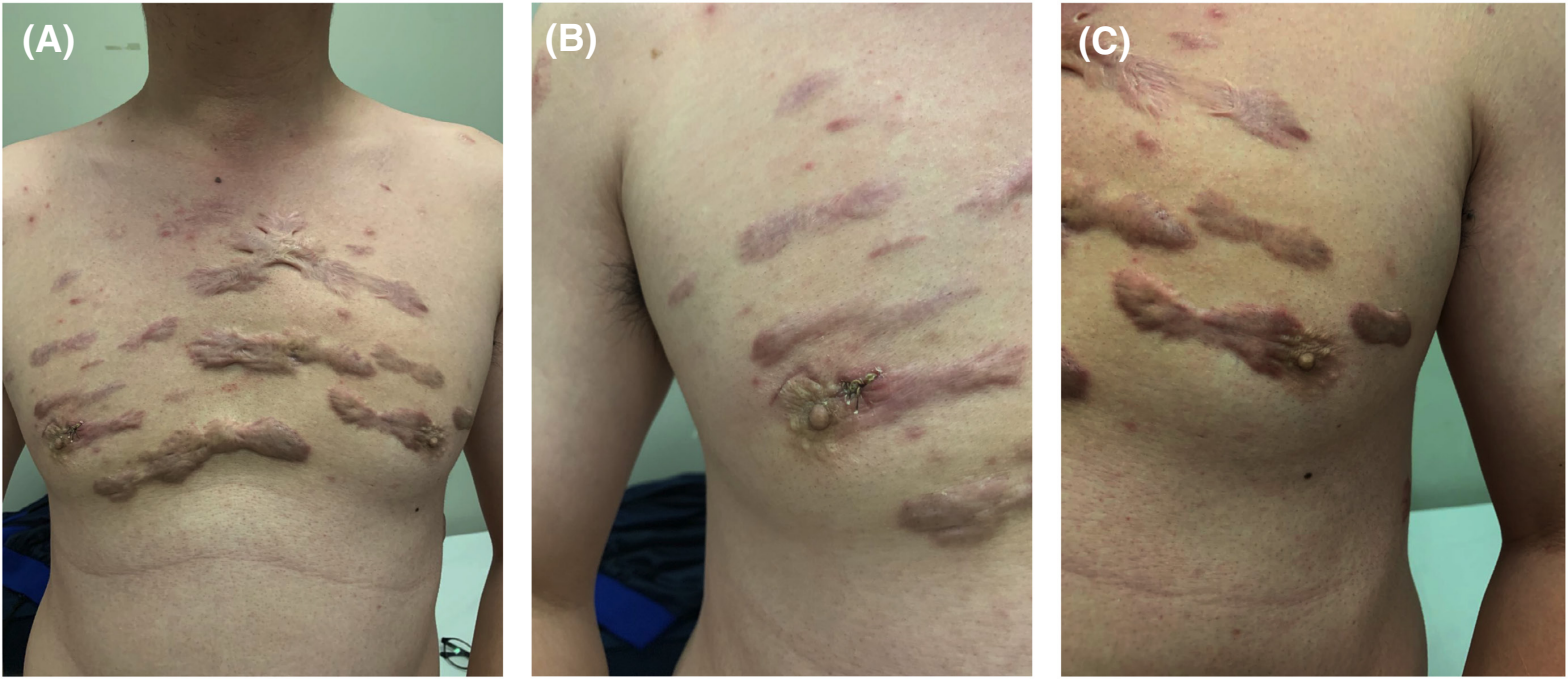
(A) The cosmetic effect of trans‐areola single‐site endoscopic thyroidectomy in patients with scar diathesis. (B, C) Comparison between surgical incision and normal areola.

In this study, operation time was used as major indicator for surgical performance and proficiency. Meanwhile, the number of retrieved LNs, postoperative complications, length of hospital stay, and surgical stress were also evaluations of surgical improvements throughout the full series. Based on CUSUM analysis, the curve peaked at case number 41 (Figure 3). The maximum turning point divided the full series into two phases: Phase 1 determined by the first 41 cases as skill acquisition period and Phase 2 determined by the remaining 181 cases as proficiency period. Operation time improved from 154.63 ± 52.21 min (Phase 1) to 95.64 ± 22.96 min (Phase 2). Comparisons of patient characteristics, surgical and oncologic outcomes, surgical stress, and postoperative complications were made between periods. Age, sex ratio, BMI, and lesion side distribution did not differ significantly between the two phases (*p* > 0.05, Table 1). In Phase 1, the benign and malignant nodule had a mean size of 2.48 ± 0.34 cm and 0.78 ± 0.51 cm, and 19.5% (8 of 41) of cases were multifocal. Meanwhile, in Phase 2, the benign and malignant nodule size was 2.70 ± 0.58 cm and 0.62 ± 0.50 cm, respectively, with 9.9% (18 of 181) of cases being multifocal. The size and number of tumor foci showed no significant differences between the two periods (*p* = 0.130, *p* = 0.111, *p* = 0.085, Table 1). Eleven patients in Phase 1 and 27 patients in Phase 2 were indicated for drainage placement. Among the 11 cases in Phase 1, the drainage amount was 29.09 ± 17.58 mL, and the total placement time was 1.45 ± 0.52 days, which were not significantly different from those in the Phase 2 (33.22 ± 14.35 mL, *p* = 0.456; 1.81 ± 0.56 days, *p* = 0.074, Table 2). Additionally, the length of postoperative hospitalization in Phase 2 was 3.65 ± 0.63 days, significantly shorter than 4.12 ± 0.93 days in Phase 1 (*p* < 0.001, Table 2). For patients who present scar diathesis, TASSET yielded excellent advantage and the incisions were almost invisible postoperatively (Figure 4).

Further analyses showed that there were no significant differences in pathological composition between the two periods (*p* = 0.159, Table 2). In detail, in Phase 1 eight cases were benign nodule and 33 cases were PTC. Consistently, there were 62 benign cases, 118 PTC and one FTC patients in Phase 2. As to TNM staging of malignant cases, 31 cases were stage I and two cases were stage II in Phase 1. Respectively, 116 patients had stage I and three patients had stage II in Phase 2 (*p* = 0.297, Table 2). The count of total LNs and metastatic LNs retrieved were 3.15 ± 1.70 (range 2–8) and 1.00 ± 1.15 (range 0–4) in Phase 1. In Phase 2, a total of 3.14 ± 1.78 (range 2–10) LNs were retrieved, with 1.06 ± 1.43 (range 0–7) being metastatic LNs. No significant differences were noticed in these numbers between periods (*p* = 0.980, *p* = 0.806, Table 2).

### 3.7 Possible impacts on learning curve

**FIGURE 5:**
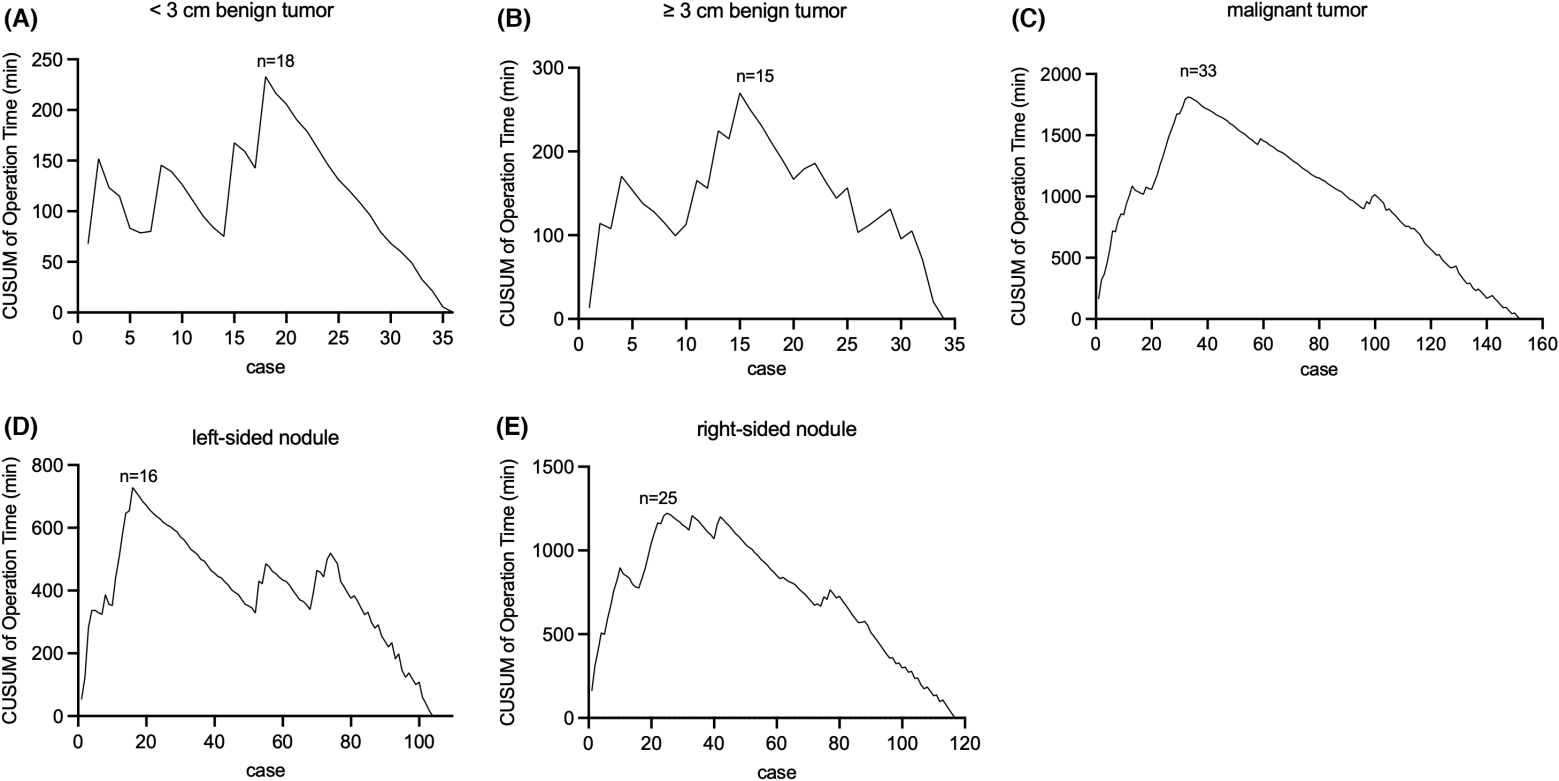
The cumulative sum (CUSUM) curve of operation time for the subgroups. (A) For <3 cm benign tumor, the turning point is at Case 18. (B) For ≥3 cm benign tumor, the turning point is at Case 15. (C) For malignant tumor, the turning point is at Case 33. (D) For left‐sided nodule, the turning point is at Case 16. (E) For right‐sided nodule, the turning point is at Case 25.

In benign cases, nodules less than 3 cm were included in the early learning stage, and no more limitation in tumor size after proficiency stage. Among 36 cases of nodule size <3 cm, the turning point was at the 18th case and the mean operation time in proficiency period was 90.61 ± 3.26 min (Table 5, Figure 5A). Based on that, another 15 cases of experience were necessary for technical proficiency in nodule size ≥3 cm group. The mean operation time could be decreased from 122.40 ± 38.97 min to 90.21 ± 21.26 min (Table 5, Figure 5B). Therefore, although the size of benign nodules had no significant impact on the lengthy of study period (*p* = 0.622, Table 6), experience from smaller nodules should be emphasized.

**TABLE 5:** The operation time of learning curve.

	Total	Skill acquisition period	Proficiency period	*p*
Full series (mean ± SD, min)	106.54 ± 38.07	154.63 ± 52.21	95.64 ± 22.96	<0.001
Benign tumor				
<3 cm nodule (mean ± SD, min)	103.56 ± 33.38	116.50 ± 43.92	90.61 ± 3.26	0.023
≥3 cm nodule (mean ± SD, min)	104.41 ± 33.97	122.40 ± 38.97	90.21 ± 21.26	0.009
Malignant tumor (mean ± SD, min)	107.72 ± 40.07	162.61 ± 50.95	92.50 ± 16.56	<0.001
Left‐sided nodule (mean ± SD, min)	102.86 ± 34.41	148.38 ± 48.52	94.58 ± 23.36	<0.001
Right‐sided nodule (mean ± SD, min)	109.78 ± 40.89	158.64 ± 55.03	96.65 ± 22.65	<0.001

**TABLE 6:** The end‐point of learning curve.

	Total	Skill acquisition period	Proficiency period	*p*
				<0.001
Benign tumor (*n*)	36	18	18	
Malignant tumor (*n*)	152	33	119	
Benign group (*n*)				0.622
<3 cm nodule	36	18	18	
≥3 cm nodule	34	15	19	
				0.266
Left‐sided nodule (*n*)	104	16	88	
Right‐sided nodule (*n*)	118	25	93	

In malignancy, 33 cases were needed to reach the initial learning stage and the mean operation time in proficient cases was 92.50 ± 16.56 min (Table 5, Figure 5C). The case number required for proficiency phase in benign and malignant tumor were 18 and 33, respectively. These results indicated that LN resection posed a significant impact on the endpoint of the learning curve (*p* < 0.001, Table 6).

Since harmonic scalpel was anchored on the right side of endoscope in TASSET, view line, and operating line was fixed and paralleled. Right and left gland resection required different surgical technique. Meanwhile, considering the different anatomical positions of left and right RLN, the time spent in identifying RLN might vary. All patients were then divided into left‐sided group and right‐sided group according to nodule location. In left‐sided group, the mean operation time of 104 patients was 102.86 ± 34.41 min. In right‐sided group, 118 patients were included and the mean operation time was 109.78 ± 40.89 min. The turning point of left‐sided group and right‐sided group were Case 16 and Case 25, which no significant difference reached (*p* = 0.266, Table 6, Figure 5D,E).

### 3.8 Influence on surgical stress and complications during different learning stage

**FIGURE 6:**
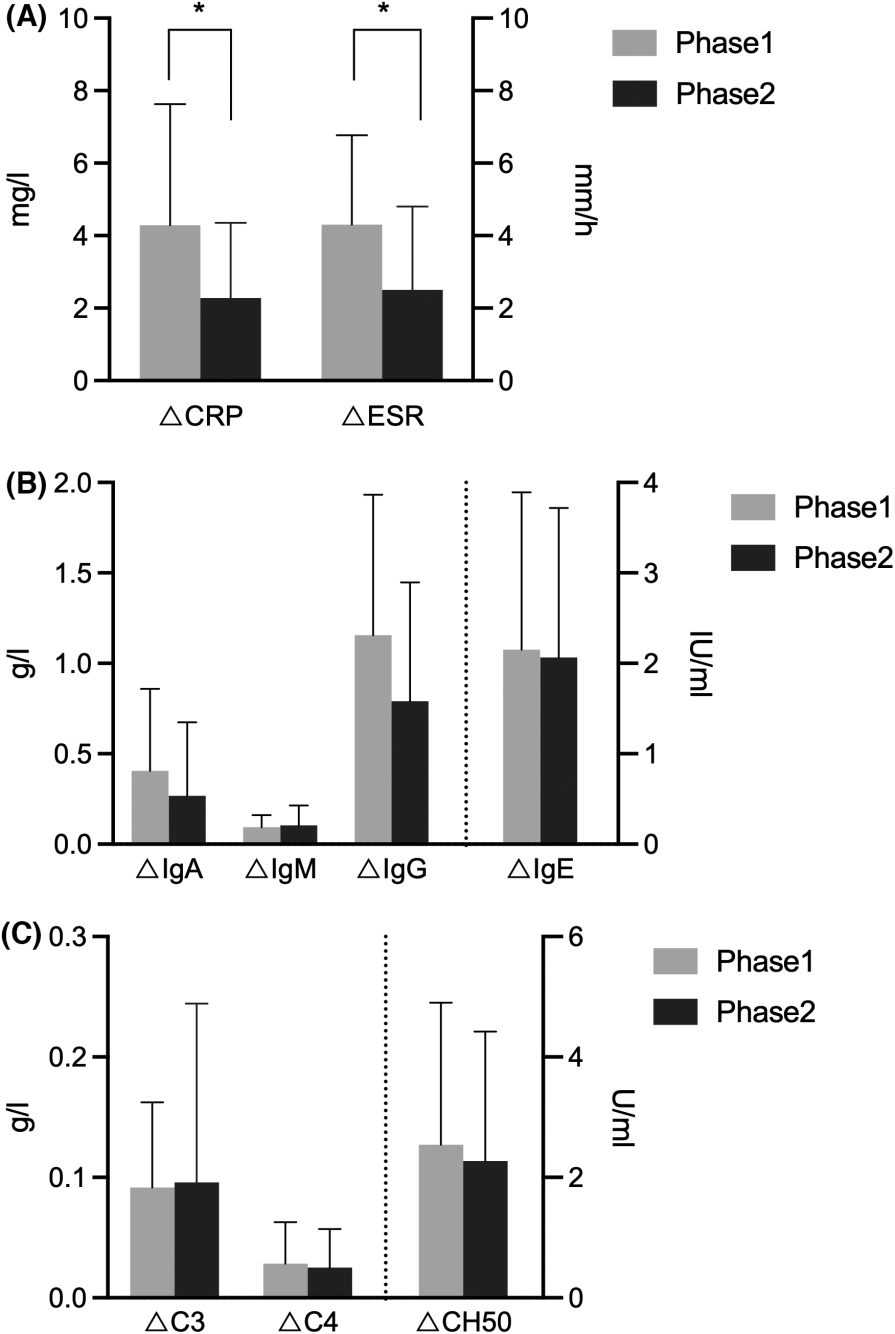
Comparisons of surgical stress outcomes between skill acquisition period (Phase 1) and proficiency period (Phase 2). (A) Improvements of stress parameters. A significant decrease was observed both in variations of C‐reactive protein (CRP) and erythrocyte sedimentation rate (ESR) between the two periods. Accumulated surgical technique would help relieve surgical trauma. (B) Changes of immunological parameters. Downtrends could be observed in variations of IgA, IgG and IgE. (C) Changes of complements. Downtrends could be observed in variation of CH50.

Regarding the traumatic index, the mean variations of traumatic parameters (CRP and ESR) in Phase 2 were significantly lower than those in Phase 1 (2.28 ± 2.08 vs. 4.29 ± 3.34 mg/L, *p* = 0.022; 2.50 ± 2.31 vs. 4.31 ± 2.46 mm/h, *p* = 0.017, Table 3, Figure 6A), whereas the variations of the remaining immunoglobulins and complements did not show any differences between the two periods (*p* > 0.05, Table 3, Figure 6B,C). The proficiency of the TASSET procedure would help relieve surgical stress.

Eleven patients developed transient voice hoarseness postoperatively. Among them, two cases occurred in Phase 1 and the recovery duration was 3.00 ± 1.41 months; nine cases occurred in Phase 2 with a recovery time of 3.22 ± 1.20 months. There were no significant differences in case number (*p* > 0.999, Table 4) and recovery time (*p* = 0.822, Table 4). Additionally, one case of permanent vocal cord palsy was noticed in either period due to unidentifiable RLN adhesion (*p* = 0.336, Table 4). No cases of hypocalcemia occurred in either period. Three and nine cases of postoperative flap seroma or hematoma occurred in Phase 1 and Phase 2, respectively, with no significant difference (*p* = 0.828, Table 4). Furthermore, in Phase 1 one case was complicated with flap burning in the anterior neck area, and this complication was absent in Phase 2 (*p* = 0.185, Table 4).

## 4 DISCUSSION

Several endoscopic techniques, including single‐site approach, have been proposed that could be potential minimally invasive substitute for open thyroidectomy.^2^, ^7^, ^13^, ^31^ With continuous understanding and mastery of single‐site approach technique, its application is increasingly extended in the field of minimally invasive surgery, such as in lung cancer surgery, colon cancer surgery, and gynecological surgery.^32^, ^33^, ^34^, ^35^, ^36^, ^37^, ^38^, ^39^, ^40^ The anatomy of chest wall is different from that of abdominal cavity, which means less trocar intubations consequently lead to less collateral injuries. Therefore, the major advantage of trans‐areola single‐site approach is to minimize the surgical burden from multiple‐site approaches, in addition to invisible neck scars.^13^ From our experience, TASSET has already demonstrated safe and technically feasible with comparable oncological outcomes to conventional open procedures. However, the possible prolonged learning stage seems to be the main obstacle for the practicality of TASSET. To solve this issue, we tried to establish the standardized procedures of TASSET through our large‐scale experience. This article exactly aimed to introduce the learning curve and to promote TASSET to a wider range of medical centers so that patients can have more options. Moreover, to the best of our knowledge, no other study but this one is conducted to quantify the learning curve of TASSET in terms of operation time and surgical stress.

Learning curve refers to the procedure of continuous learning and repeated training for a certain skill.^41^ Experience from a certain number of cases was required for getting used to the down‐to‐up visual and surgical manipulation directions of TASSET approach. The identification of the end‐point learning curve could provide data for surgeons who would like to attempt TASSET and avoid complicated cases in skill acquisition period. Operation time was considered as main index of surgical proficiency.^42^ Additionally, the number of LNs retrieved and the incidence of surgical complications should also be taken into consideration. In this study, there were no significant differences in demographic data, nodule size, and laterality between periods. Therefore, we indicated that improvements in operation time were results of mastery of surgical procedure. A significant decrease in postoperative hospitalization after the end‐point learning curve was also noted as the accumulated surgical experience.

Among standardized TASSET procedures, creating working space is the main time‐consuming step in Phase 1, especially for those with higher BMI. Once the learning curve was passed, great progress could be achieved for operative time. RLN recognition and protection was another speed‐limiting step. RLN injury was among the top serious complications in thyroid surgery and proficient visual identification of RLN could be the key to prevention. Through our experience, due to the fixed right‐handed relationship between view line and operating line, the right side RLN could be found at the top artery side of the angle between the inferior thyroid artery and tracheoesophageal groove. Whereas this fixed relationship required different maneuver for the left side RLN exploration, which could be found on the esophageal side of inferior thyroid artery and the surface of tracheoesophageal fascia.^43^ Considering the differences in anatomy and exploration direction, 16 cases were needed to reach the proficient stage in left‐sided TASSET, which was slightly quicker than that of the right‐sided TASSET (25 cases), though the difference was not statistically significant. Therefore, in single‐site surgical setting, our standard procedures could narrow the gap between tumor location and manipulate position.

According to learning curve, operation time was gradually stabilized around 95 min after 41 cases. Compared with benign tumor group, 15 more cases were needed to reach the mastery level of surgical skill in malignant tumor group due to the additional CND procedure. The CND procedure is one of the challenging aspects of TASSET. TASSET only has one surgical apparatus in the surgical field all the time. The technical challenges of CND via TASSET were usually caused by collisions between the apparatus and endoscope, poor exposure of center compartment, and lack of manipulation triangle. The application of needle retractors and mini‐clamps can provide more flexibility to grasp, compress, separate, and stretch the tissue. All these maneuvers assist in establishing the triangulation space with strap muscle retraction for better exposure, which are very important for the radical dissection of the lymph nodes, especially the lymph nodes located under the common carotid artery near the RLN. Moreover, the increased flexibility also helps decrease the difficulty of operation and the time to recognize this procedure.

Surgeon's prior experience of endoscopic surgery probably poses an impact on operation. The surgeon in this study has experience from 10 to 20 cases multiple‐site endoscopic thyroidectomy, which may have a latent priority. As for surgeons who have no experience of endoscopic surgery, the learning stage would probably be relatively prolonged. Therefore, we recommended beginners to start with benign tumor cases in the initial learning stage. Meanwhile, the frequency of surgery is also one of the factors that affects the learning curve.^44^, ^45^, ^46^, ^47^ In this study, fixed number of TASSET in fixed surgery day ruled out this influence to the learning curve.

The current study has several limitations. First, larger sample‐size prospective and multicenter studies are needed to further validate our findings. Second, bilateral thyroidectomy was excluded in our series to remain data homogeneity, the learning curve of which is still under investigation. Third, cumulatively analyzing the learning curve with additional variables are warranted, such as Hashimoto thyroiditis and Graves' disease also need further investigation.

## 5 CONCLUSIONS

TASSET has demonstrated safe and technically feasible with comparable oncological outcomes. Experience of 41 cases was required for surgical competence and proficiency. The initial learning stage could be more quickly adopted by high‐volume thyroid surgeons with standardized procedures.

## AUTHOR CONTRIBUTIONS

**Ling Zhan:** Conceptualization (lead); data curation (equal); writing – original draft (equal). **Ming Xuan:** Writing – review and editing (equal). **Hao Ding:** Writing – review and editing (equal). **Juyong Liang:** Writing – review and editing (supporting). **Qiwu Zhao:** Formal analysis (supporting). **Lingxie Chen:** Formal analysis (supporting). **Zheyu Yang:** Formal analysis (supporting). **Xi Cheng:** Formal analysis (supporting). **Jie Kuang:** Formal analysis (supporting). **Jiqi Yan:** Formal analysis (supporting). **Wei Cai:** Supervision (supporting). **Weihua Qiu:** Funding acquisition (equal); supervision (supporting).

## FUNDING INFORMATION

This study was supported by the National Natural Science Foundation of China (No. 82072948).

## CONFLICT OF INTEREST STATEMENT

The authors report no conflicts of interest. The authors are responsible for the content and writing of the paper.

## Data Availability

The data that support the findings of this study are available on request from the corresponding author. The data are not publicly available due to privacy or ethical restrictions.
